# Expression of the *Foraging* Gene Is Associated with Age Polyethism, Not Task Preference, in the Ant *Cardiocondyla obscurior*

**DOI:** 10.1371/journal.pone.0144699

**Published:** 2015-12-09

**Authors:** Jan Oettler, Anna-Lena Nachtigal, Lukas Schrader

**Affiliations:** Institut für Zoologie, Universität Regensburg, 93053, Regensburg, Germany; Universidade de São paulo, BRAZIL

## Abstract

One of the fundamental principles of social organization, age polyethism, describes behavioral maturation of workers leading to switches in task preference. Here we present a system that allows for studying division of labor (DOL) by taking advantage of the relative short life of *Cardiocondyla obscurior* workers and thereby the pace of behavioral transitions. By challenging same-age young and older age cohorts to *de novo* establish DOL into nurse and foraging tasks and by forcing nurses to precociously become foragers and *vice versa* we studied expression patterns of one of the best known candidates for social insect worker behavior, the *foraging* gene. Contrary to our expectations we found that *foraging* gene expression correlates with age, but not with the task foraging *per se*. This suggests that this nutrition-related gene, and the pathways it is embedded in, correlates with physiological changes over time and potentially primes, but not determines task preference of individual workers.

## Introduction

Age or temporal polyethism in holometabolous insect societies describes the common transition from young workers performing brood/queen care to nest duties and finally to exploration of the environment and foraging later in life. Age polyethism profoundly shapes social organization but the proximate mechanisms for its formation are unknown. It has been suggested that intrinsic physiological changes of individuals could render their behavior with age [1]. Alternatively, even though individuals may have altered physiology, individual experience and social environment may drive behavior, thus providing a system where demand and not physiology shapes behavior [2]. And indeed age is only partly predictive for worker behavior as shown in bee and ant workers that readily switch between interior and exterior tasks in response to environmental conditions (e.g. [3,4]). The switch of honey bee foragers to so called “reverted nurses” is accompanied by re-appearance of nurse-like methylation patterns which affect, among others, genes involved with regulation of transcription [5].

However, there is clear evidence for discrete hormonal differences associated with task performance. In fact, foragers of ants and bees exhibit higher juvenile hormone levels than workers performing tasks inside the nest [6,7]. Furthermore, conserved hunger- and food-related molecular pathways identified in solitary species appear to determine the age at which bee workers mature into the foraging phenotype [8]. One such example is the *foraging* (*for*) gene coding for a cyclic guanosine mono-phosphate (cGMP)-dependent kinase (PKG). The role of *for* variants has been shown to play a critical role in the regulation of foraging behavior and energy balance in flies [9,10]. *Sitter*, homozygous for the allele *for* ^s^ and *rover*, carrying the allele *for* ^r^, differ in their sensitivity to sucrose and exploration strategy. Under low nutritional conditions both alleles have their highest fitness when rare, rendering this system as one of the few clear-cut examples for frequency dependent fitness effects of a single gene [11].

Under the hypothesis that nutrient-sensitive pathways correlate with behavioral plasticity in ants, *foraging* expression has been shown to differ between morphs and behavioral types. In *Pogonomyrmex* harvester ants, the expression of the *foraging* ortholog is higher in young worker brains compared to foragers [12]. *foraging* expression in this system shows diurnal fluctuations in foragers while expression remains constant in nest workers [13]. Furthermore, *foraging* is expressed in brains of *Pheidole* major workers, but not in minor workers. Pharmacological activation of PKG in major workers increases defense behavior while reducing foraging activity [14]. Based on these correlative and experimental data PKG has been suggested to play a decisive role in division of labor (DOL) in social insects. Here, we aim to disentangle age effects, task preference and task performance to test whether *foraging* is a leader or a follower in the evolution and maintenance of DOL. We identify the *foraging* ortholog in *Cardiocondyla obscurior* (*Cofor*) and by using qPCR show that its expression is associated indirectly with age polyethism rather than being directly linked with the tasks performed.

## Methods

We used descendants of a 2009 collected population of *C*. *obscurior*, an invasive cosmotropic minute (queens: 3mm, workers 2mm) myrmicine ant that lives in trees and shrubs (permitted by the Brazilian Ministry of Science and Technology (RMX 004/02)). Colonies in the wild consist of several queens and up to a few dozen workers [15] but can reach several hundred workers in the lab. Experimental colonies were set up from these large stock colonies that are maintained in climate-controlled chambers (27°C/23°C, 12/12 light/dark). Experimental colonies were kept in plaster-floored Petri dishes (92x16mm) with a preformed cavity covered with dark foil as nest site. These colonies were kept in the same climate-controlled room where we performed the experiments at a constant 25°C and a 12 light / 12h dark cycle. Colonies were connected to a second Petri dish via a 20cm long ~3mm thick hemp thread which was set up between two poles made from toothpicks in 30mm wide and 100mm tall plaster stands. On this second petri dish we placed a 1cm small plate with diluted honey and a small piece of cockroach, which was daily renewed. In a series of preliminary experiments we determined that the ants do not use mass recruitment to food but rather forage individually, with occasional tandem-running (data available upon request). We applied a thin layer of paraffin onto the walls of both petri dishes to prevent workers from leaving the arena. Prior to the experiments we set up four colonies consisting of 20 workers and two queens. We filmed (DigiMicro 2.0 Scale, dnt), trigger recorded (Camera Security Software iSpy 4.9.1.0) and counted foragers arriving at the food over 72 hours. Because of a slight increase in activity when the lights came on at 8:00am (data available upon request) we performed all experiments between 8:00 and 12:00 am.

### Native DOL (control experiments): Young callows vs. age-unknown foragers and age-unknown nurses vs. age-unknown foragers

We set up two lines of controls using unmanipulated colonies. First, we tested whether *foraging* expression differs between young and old workers. To this end, five stock colonies were given access to a food source for 15 days, to establish a stable nurse-foraging task allocation. From each colony we then pairwise collected ten foragers drinking from the honey and ten callows (1–2 day old workers that have just finished metamorphosis, still with a soft and yellowish cuticle) that tended to a larva or egg in the nest. All samples were immediately individually snap-frozen in liquid nitrogen.

Second, to test whether behavioral groups identified only by their task preference differ, we set up five stock colonies and after 15 days pairwise collected and froze from each of the colonies five honey-drinking foragers and five workers that tended a larva or egg, indicative of nursing behavior. In this and all subsequent experiments we avoided collecting workers that responded to a disturbance by picking up brood in a frantic manner. This behavior is indicative of a response to stress caused by the experimenter and putatively is performed by all members of a colony and not only by nurse workers. Consequently, in those few cases where the experimenter obviously was not unnoticed the sampling was postponed and repeated later that day.

### Age-independent DOL: 1 week-old nurses vs. foragers (“week1”) and 5 week-old nurses vs. foragers (“week5”)

To test whether same age individuals displaying different task preference differ in *foraging* expression, we established five experimental colonies consisting of two mated queens, 25 similar aged dark worker pupae and some brood. After two days, when most pupae had eclosed, the nests were connected to the food source. After five additional days and after establishment of a primary DOL we pairwise collected five individuals engaging in foraging and five individuals performing nurse work from each colony and froze them.

This design was repeated with four colonies again consisting of two mated queens and 25 worker pupae. Once these pupae emerged as adults we removed any additionally developed pupae for the next 30 days to maintain the initial age cohort. We then connected these same-aged colonies to the food source for five days and pairwise sampled five foragers and five nurse workers from each colony.

### Coerced secondary DOL: Age-unknown reverted nurses vs. foragers and age-unknown nurses vs. precocious foragers

Finally, we manipulated task preference by initiating reverted nurses and precocious foragers. First, we connected five large stock colonies to a food source. From each colony we prepared two new experimental colonies with two adult queens, brood and five workers. After two days these workers were removed and each of the experimental colonies then received either 30 foragers or 30 nurse workers from the previously set up large stock colonies, respectively, which were identified and collected as described before. The experimental colonies consisting of either only original nurses or only original foragers were then connected to a food source, inducing an afresh DOL into nursing and foraging tasks in both types of colonies. After seven days we pairwise collected five foragers and five nurses from each colony and froze them.

To test for differences in task preference between primary foragers and precocious foragers we recorded for one hour the number of honey-drinking workers at the food source immediately after we connected the colony with the food.

### RNA extraction and qPCR

All ants were decapitated under a microscope on a pre-cooled metal dissection tray placed in liquid N2. Pools of five worker heads from each replicate were homogenized with a FastPrep (MP Biomedicals). Total RNA was extracted with an RNeasy Plus Micro Kit (Qiagen) and transcribed with an iScript cDNA synthesis kit (Bio-Rad). RT-qPCR with 5ng input cDNA per reaction in triplicates was done with a CFX connect (Bio-Rad) using Kapa Sybr Fast (Peqlab).

### Annotation of the *C*. *obscurior foraging* ortholog, primer design and statistical analysis

Fly *for* has two homologs in *C*. *obscurior*, similar to *Pheidole pallidula* [14]. *Cobs_17572* has a catalytical domain (STKc_cGK_PKG [cd05572]), two cGMP-binding domains (CAP_ED [cd00038]) and a regulatory domain (DD_cGKI-beta [cd12086]). The shorter *Cobs_15824* only has one cGMP-binding domain and lacks the catalytical domain. Similar to previous studies in ants, we focused only on the former [14,16], hereafter named *Cofor*. After manual correction of the gene model guided by draft genomes of five ant species (*Camponotus floridanus* EFN63550.1, *Harpegnathos saltator* EFN87545.1, *Solenopsis invicta* EFZ12468.1, *Pogonomyrmex barbatus* AAV65146.1, and *Linepithema humile LH17994*), the cloned and Sanger-sequenced *P*. *pallidula Ppfor* T1 protein sequence, *Apis mellifera* (NP_001011581.1) and *Drosophila* (ACO44435.1), we designed a primer pair for a 143bp amplicon spanning the 1^st^ and 3^rd^ exon to avoid amplification of possible genomic DNA contamination (for_LS_T1: forward: TGGTGAAGTTCCCGAAGCCGCA; reverse: CGAGCCAGCTGGAAATGTAACGGG). We used a single housekeeper, *rps2* (*Cobs_18295*) because of limited input cDNA of the samples (rps2: forward: AAGCCATTCTGCGATGGCC; reverse: TCGAAGCCAACATGCTTAGCG). A serial dilution starting with 50ng input cDNA showed similar efficiencies with 92.5% for *Cofor* and 87.8% for rps2, respectively.

For interpretation of the qPCR data we used the relative 2^-ΔCq^ [17] method, followed by a Mann-Whitney-U test within treatments in R (“wilcox.test”).

## Results

**Fig 1:**
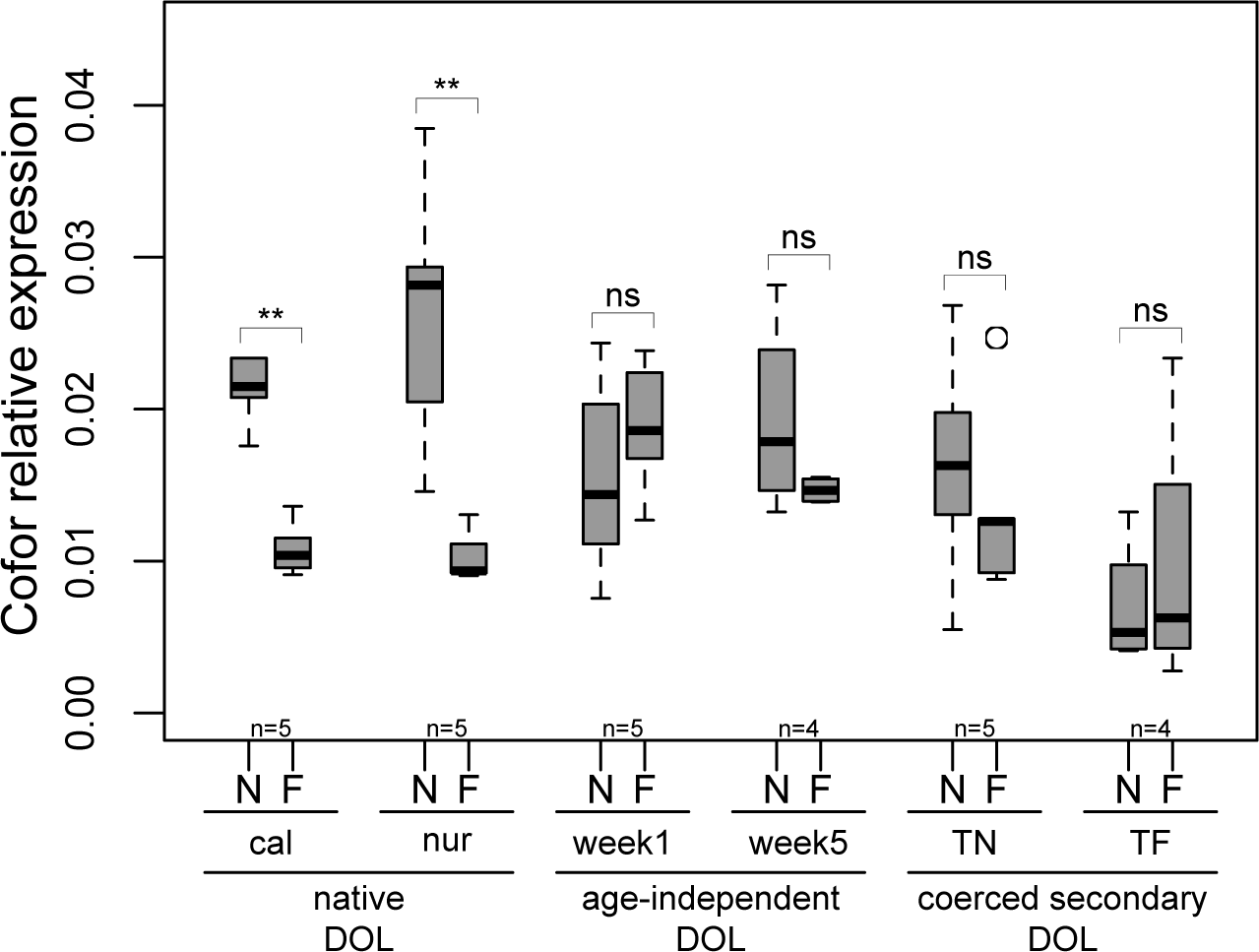
Relative *Cofor* expression comparing nurses (N) and forager (F) expression. “cal” = callows performing nursing tasks vs. foragers; “nur” = age-unknown nurses vs. foragers; “week1” = 1 week-old nurses vs. foragers; “week5” = 5 week-old nurses vs. foragers; “TN” = Age-unknown workers engaged in the task nursing and challenged to precocious foraging behavior; “TF” = Age-unknown workers engaged in the task foraging and challenged to revert to nursing behavior; DOL = division of labor. ** = p<0.008, ns = p>0.05. The number of replicate colonies is given below each box; each replicate represents five pooled nurse and worker heads, respectively.

Both control experiments (native DOL) showed the predicted direction of differential expression with callows engaged in nursing behavior (W = 25, p<0.008, Fig 1) and also nurses of unknown age (W = 25, p<0.008, Fig 1) having higher relative levels of *Cofor* expression compared to age-unknown foragers, respectively. In contrast, neither of the two age treatments (age-independent DOL) showed significant differences in *foraging* expression between task groups (1 week old: W = 9, p = 0.548; 5 week old: W = 12, p = 0.343, Fig 1). Furthermore, there was no difference in *Cofor* expression between task groups in the experiment challenging nurses to become precocious foragers (TN: W = 0.17, p = 0.421) and between task groups in the experiment challenging foragers to become reverted nurses (TF: W = 7, p = 0.886, Fig 1, secondary coerced DOL).

After establishment of the latter two experiments we compared the numbers of individuals displaying foraging behavior during the first hour of the experiment. As predicted, nurses that were challenged to become precocious foragers foraged less in the very first hour of the experiment compared to primary foragers (t-Test: t = 2.6224, p<0.031; foragers mean ± SD: 42.00 ± 11.68, nurses: 22.40 ± 11.95).

## Discussion

In line with previous studies on ants [12,14], control callows and young nurses similarly had relative higher *Cofor* expression levels than foragers, suggesting that *C*. *obscurior* follows an age-related behavioral trajectory with young nurses and older foragers. In social species without specialized morphological worker castes it has been suggested that old, disposable workers with low body-fat perform those exterior tasks with high mortality risks [18]. Gene expression of fly *for* variants partly correlates with the insulin-signalling pathway, which responds to hemolymph blood sugar levels. Similarly, *Cofor* likely plays a role in nutrient-dependent physiological processes in *C*. *obscurior*, and its expression could relate to body-fat and carbohydrate levels, which are predicted to be higher in young workers.

While *Cofor* expression in control colonies clearly shows a correlation with behavioral phenotype, regardless whether nurses were less than two days old or age-unknown, there was no correlation of *Cofor* expression with task performance per se. Furthermore, workers readily switched tasks in the manipulated colonies, regardless of whether they were young or matured, or already performed a particular task previously. This implies that the absence of successful returning foragers or signals from hungry brood motivates individual workers to switch task and to revert to nursing or to become precociously explorative, independent of *Cofor* expression.

In general, the various contexts in which this gene is expressed in different taxa, genotypes, morphs, behavioral types, age stages, day times and cells, as well as conflicting expression direction between ants and bees [19], shows that this gene is involved in fundamental physiological plasticity. This study adds to our understanding of this genes’ involvement in plasticity. Together, our findings suggest that *foraging* could function in priming individuals for a particular task but that it is not indispensible for the establishment of DOL in social insects. It may thus be considered a textbook example for a gene that is a follower in many evolutionary processes [20–22] and evolved to be a leader in one [11].

Ant workers appear to be capable of the full behavioral repertoire once they reach a certain age, although the genetic background in polyandric species can play a role in individual task preference [23,24] and colony-level foraging performance [25]. A less deterministic interpretation of individual and colony level personalities [26,27] similarly regards the social environment as the ultimate driver of behavioral plasticity, and its underlying genetic correlates are suggested to lie in reversible gene regulation differences [5]. The *C*. *obscurior* system with an established draft genome [15] and first comparative neuroanatomic resources [28] in combination with simple behavioral manipulations of complete colonies—such as the ones presented here—will allow for better understanding of the biology of individual worker personalities and division of the non-reproductive labor.

## Supporting Information

S1 Data RT-qPCR raw Cq values. (ZIP)

## References

[1] Robinson GE. Regulation of division of labor in insect societies. Annu Rev Entomol. 1992;37: 637–665. 10.1146/annurev.en.37.010192.003225 1539941

[2] Franks NR, Tofts C. Foraging for work—How tasks allocate workers. Anim Behav. 1994;48: 470–472. 10.1006/anbe.1994.1261

[3] Leoncini I, Le Conte Y, Costagliola G, Plettner E, Toth AL, Wang MW, et al Regulation of behavioral maturation by a primer pheromone produced by adult worker honey bees. Proc Natl Acad Sci U S A; 2004;101: 17559–17564. 10.1073/pnas.0407652101 15572455 PMC536028

[4] Oettler J, Johnson RA. The old ladies of the seed harvester ant *Pogonomyrmex rugosus*: foraging performed by two groups of workers. J Ins Behav. 2008;22: 217–226. 10.1007/s10905-008-9167-7 PMC3085757 21654914

[5] Herb BR, Wolschin F, Hansen KD, Aryee MJ, Ben Langmead, Irizarry R, et al Reversible switching between epigenetic states in honeybee behavioral subcastes. Nature Neuroscience; 2012;15: 1371–1373. 10.1038/nn.3218 22983211 PMC3518384

[6] Amdam GV, Norberg K, Hagen A, Omholt SW. Social exploitation of vitellogenin. Proc Natl Acad Sci U S A. 2003;100: 1799–1802. 10.1073/pnas.0333979100 12566563 PMC149913

[7] Dolezal AG, Brent CS, Hölldobler B, Amdam GV. Worker division of labor and endocrine physiology are associated in the harvester ant, *Pogonomyrmex californicus* . J Exp Biol. 2012;215: 454–460. 10.1242/jeb.060822 22246254 PMC3257171

[8] Ament SA, Corona M, Pollock HS, Robinson GE. Insulin signaling is involved in the regulation of worker division of labor in honey bee colonies. Proc Natl Acad Sci U S A. 2008;105: 4226–4231. 10.1073/pnas.0800630105 18337502 PMC2393790

[9] Kent CF, Daskalchuk T, Cook L, Sokolowski MB, Greenspan RJ. The *Drosophila* foraging gene mediates adult plasticity and gene-environment interactions in behaviour, metabolites, and gene expression in response to food deprivation. PLoS Genet. 2009;5: e1000609 10.1371/journal.pgen.1000609 19696884 PMC2720453

[10] Kaun KR, Sokolowski MB. cGMP-dependent protein kinase: linking foraging to energy homeostasis. Genome. 2009;52: 1–7. 10.1139/G08-090 19132066

[11] Fitzpatrick MJ, Feder E, Rowe L, Sokolowski MB. Maintaining a behaviour polymorphism by frequency-dependent selection on a single gene. Nature. 2007;447: 210–212. 10.1038/nature05764 17495926

[12] Ingram KK, Oefner P, Gordon DM. Task-specific expression of the foraging gene in harvester ants. Mol Ecol. 2005;14: 813–818. 10.1111/j.1365-294X.2005.02450.x 15723672

[13] Ingram KK, Kleeman L, Peteru S. Differential regulation of the foraging gene associated with task behaviors in harvester ants. BMC Ecology; 2011;11: 19 10.1186/1472-6785-11-19 21831307 PMC3180247

[14] Lucas C, Sokolowski MB. Molecular basis for changes in behavioral state in ant social behaviors. Proc Natl Acad Sci U S A. 2009;106: 6351–6356. 10.1073/pnas.0809463106 19332792 PMC2669355

[15] Schrader L, Kim JW, Ence D, Zimin A, Klein A, Wyschetzki K, et al Transposable element islands facilitate adaptation to novel environments in an invasive species. Nature Communications. 2014;5: 5495 10.1038/ncomms6495 25510865 PMC4284661

[16] Lucas C, Kornfein R, Chakaborty-Chatterjee M, Schonfeld J, Geva N, Sokolowski MB, et al The locust foraging gene. Arch Insect Biochem Physiol. 2010;74: 52–66. 10.1002/arch.20363 20422718

[17] Schmittgen TD, Livak KJ. Analyzing real-time PCR data by the comparative CT method. Nat Protoc. 2008;3: 1101–1108. 10.1038/nprot.2008.73 18546601

[18] Porter SD, Jorgensen CD. Foragers of the harvester ant, *Pogonomyrmex owyheei—*a disposable caste. Behav Ecol Sociobiol. 1981;9: 247–256. 10.1007/BF00299879

[19] Ben-Shahar Y, Robichon A, Sokolowski MB, Robinson GE. Influence of Gene Action Across Different Time Scales on Behavior. Science; 2002;296: 741–744. 10.1126/science.1069911 11976457

[20] West-Eberhard MJ. Developmental plasticity and the origin of species differences. Proc Natl Acad Sci U S A. 2005;102 Suppl 1: 6543–6549. 10.1073/pnas.0501844102 15851679 PMC1131862

[21] Schwander T, Leimar O. Genes as leaders and followers in evolution. Trends in Ecology & Evolution. 2011;26: 143–151. 10.1016/j.tree.2010.12.010 21257223

[22] Uller T, Helanterä H. When are genes ‘leaders’ or ‘followers’ in evolution? Trends in Ecology & Evolution. 2011;26: 435–436. 10.1016/j.tree.2011.05.013 21715043

[23] Julian GE, Fewell JH. Genetic variation and task specialization in the desert leaf-cutter ant, *Acromyrmex versicolor* . Anim Behav. 2004;68: 1–8. 10.1016/j.anbehav.2003.06.023 10458895

[24] Waddington SJ, Santorelli LA, Ryan FR, Hughes WOH. Genetic polyethism in leaf-cutting ants. Behav Ecol. 2010;21: 1165–1169. 10.1093/beheco/arq128

[25] Wiernasz DC, Hines J, Parker DG, Cole BJ. Mating for variety increases foraging activity in the harvester ant, *Pogonomyrmex occidentalis* . Mol Ecol. Blackwell Publishing Ltd; 2008;17: 1137–1144. 10.1111/j.1365-294X.2007.03646.x 18261053

[26] Holbrook CT, Wright CM, Pruitt JN. Individual differences in personality and behavioural plasticity facilitate division of labour in social spider colonies. Anim Behav. 2014;97: 177–183. 10.1016/j.anbehav.2014.09.015

[27] Modlmeier AP, Liebmann JE, Foitzik S. Diverse societies are more productive: a lesson from ants. Proc Royal Soc B. 2012;279: 2142–2150. 10.1098/rspb.2011.2376 PMC3321703 22279166

[28] Bressan JMA, Benz M, Oettler J, Heinze J, Hartenstein V, Sprecher SG. A map of brain neuropils and fiber systems in the ant *Cardiocondyla obscurior* . Front Neuroanat.; 2014;8: 166 10.3389/fnana.2014.00166 25698935 PMC4316776

